# An Overview of the Neglected Modes of Existence in Avian Haemosporidian Parasites

**DOI:** 10.3390/microorganisms13050987

**Published:** 2025-04-25

**Authors:** Gediminas Valkiūnas, Tatjana Iezhova

**Affiliations:** State Scientific Research Institute Nature Research Centre, Akademijos 2, 08412 Vilnius, Lithuania

**Keywords:** *Plasmodium*, *Haemoproteus*, birds, persistence, life cycle, gametocytes, merogony

## Abstract

Haemosporidian parasites (Apicomplexa, Haemosporida) are diverse obligatory heteroxenous protists, which infect all major groups of terrestrial vertebrates and use dipterous blood-sucking insects as vectors. These pathogens are responsible for various diseases, including malaria, which remains an important human and animal illness. In the wild, haemosporidians are particularly diverse in reptiles and birds in tropical countries, where they are flourishing. Avian haemosporidians have been particularly extensively investigated, especially due to their high prevalence and global distribution, including the countries with cold climates. The general scheme of the life cycle of haemosporidians is known, but the details of development remain insufficiently investigated or even unknown in most of the described parasite species, suggesting the existence of knowledge gaps. This attracts attention to some recent observations, which remain fragmentary but suggest the existence of formerly neglected or underestimated modes of the haemosporidians’ survival in vertebrates. Such findings are worth discussion as they indicate the novel directions in wildlife haemosporidian research. This article overviews some recent findings, which call for broadening of the orthodox views on modes of existence of haemosporidian parasites in avian hosts. Among them are the role of blood merogony in the long-lasting persistence of malaria parasites in birds, the role of gametocytes in the long-lasting survival of *Haemoproteus* species in vertebrates, the possible reasons of undetectable avian *Haemoproteus* infections due to peculiarities of exo-erythrocytic development, and the plausible factors driving the narrow vertebrate host specificity of *Haemoproteus* species.

## 1. Introduction

Wildlife haemosporidian parasites or haemosporidians (Apicomplexa, Haemosporida) have been subjects of extensive research after their discovery in birds at the end of 19th century [1]. The scientific interest in these organisms has been permanently increasing due to their close relationships with human malaria pathogens, widespread distribution, readily available templates for DNA analysis using unlimited samples from live wild animals, insufficiently investigated pathology, and evolutionary issues [2]. Recently, the avian haemosporidians have attracted attention as model organisms for the investigation of their unique associations with Matryoshka RNA viruses, which probably use these cosmopolitan parasites as vectors for transmission between vertebrates and blood-sucking insects [3,4].

Haemosporidians are diverse obligatory heteroxenous protists with important similarities in their life cycles, physiology, and reproduction. They are currently grouped in four families—Plasmodiidae, Haemoproteidae, Leucocytozoidae, and Garniidae—with relatively well-summarised knowledge on their life cycles, of which the general outline is as follows [2,5]. Parts of the life cycle occur within blood-sucking dipterous vectors (final hosts) and parts occur within vertebrates (intermediate hosts). Sporozoites (infective for vertebrates stage) are formed in oocysts, which develop in vectors. In vertebrates, asexual multiplication occurs and develop intracellular gametocytes (infective stage for vectors). Birds become infected when vectors release sporozoites into susceptible hosts during a blood meal. The sporozoites initiate asexual multiplication (merogony) in cells of fixed tissues and develop into tissue meronts (small tissue stages) or megalomeronts (large tissue stages), in which merozoites develop. The latter are stages responsible for the spread of infection within the vertebrate hosts. Some merozoites from tissue stages invade blood cells and develop into sexual stages (gametocytes), while others continue merogony in the circulating blood cells (only in species of Plasmodiidae and some Garniidae parasites) or initiate new generations of tissue stages. Once infected with haemosporidians, birds usually remain infected for many years or even for their lifespan; however, the mechanisms of the long-lasting persistence remain insufficiently understood [5,6].

Thousands of publications are available on various aspects of the biology of wildlife haemosporidians [5,7,8,9,10,11,12,13,14,15], and several recent reviews described progress in understanding of their biology [2,13,16,17,18]. Life cycles of human *Plasmodium* species are particularly deeply investigated; however, some controversial gaps remain even in the understanding of delicate development of the well-known malaria pathogen *Plasmodium falciparum*, for example, the mechanisms of asexual growth [16]. Knowledge on the peculiarities of development of haemosporidians inhabiting wildlife animals remain strikingly fragmentary or even non-accessed in the great majority of described species, suggesting the existence of possible knowledge gaps [13,17,18,19].

The application of molecular diagnostic methods added prominent information about the genetic aspects of biology of haemosporidians in wildlife but often remains insufficiently powerful in unravelling modes of their existence in nature. For example, the currently applied methodologies of molecular detection of a parasite DNA readily shows the presence of the pathogen in hosts but often tell little about biological meaning of the detected genetic signals regarding their significance in the life cycle and transmission [2,17,19]. This is a particularly sensitive issue due to the common abortive haemosporidian infections in wildlife [20,21,22,23,24]. The latter occur both in blood-sucking insects and vertebrates if the parasite’s invasive stages reach either unsusceptible or partially susceptible hosts, in which development is arrested but the template for PCR-based detection remains, resulting in positive DNA amplifications and detection of genetic lineages [2,20,21,22,23,24]. However, the positive PCR-based signals do not necessarily indicate that the hosts can support the complete development and the production of invasive stages, which are essential for the infection spread. Such information might mask the true picture about the range of competent avian hosts and vectors, resulting in difficulties understanding the epidemiological situation in nature. Delicate experimental observations, which combine laboratory-controlled research and molecular genetics are needed, but such studies remain rare in wildlife, particularly due to the restrictions to use wild vertebrates in experimental academy observations. The latter issue is understandable from the point of view of wildlife care; however, it markedly slows down research on the biology of wildlife pathogens and seems difficult to overcome in the near future due to the global wildlife protection policy. This remarkably increases the value of some recent field and experimental observations, which remain fragmentary but suggest the existence of formerly neglected or underestimated features of haemosporidian existence in wildlife. Such findings are worth attention as the indications of possible novel directions in haemosporidian research. This study aimed to overview some recent findings, which are challenging and suggest broadening of some orthodox views on modes of existence of wildlife haemosporidian parasites.

## 2. Materials and Methods

Full-length papers published in peer-reviewed journals were considered. Articles have been retrieved from online bibliographic databases PubMed, SCOPUS, and Google Scholar. Published bibliographies of the avian blood-inhabiting haematozoa were also used [5,7,8,9,10,11,12]. Histological preparations of exo-erythrocytic stages of *Haemoproteus*, *Plasmodium*, and *Hepatocystis* species were obtained from the collections of State Scientific Research Institute Nature Research Centre (Vilnius, Lithuania), International Reference Centre for Avian Haematozoa (Queensland Museum, South Brisbane, Queensland, Australia), Natural History Museum (London, UK), and the US National Parasite Collection (National Museum of Natural History, Washington, DC, USA). In all, over 200 histological sections of exo-erythrocytic stages were examined.

Blood films from birds sampled at Ventė Cape (55°20′27″ N, 21°11′22″, Ventės ragas Ornithological station, Lithuania) in May 2015–2019 and 2021–2024 and deposited in State Scientific Research Institute Nature Research Centre were the main source of data for estimation of parasitaemia. The study site was described in [25]. In all, 134 bird individuals belonging to eight species naturally infected with single infections of *Haemoproteus* parasites were used in estimation of synchronous parasitaemia occurrence during this study (see the Results for further explanation). Among them were 38 individuals of *Phylloscopus trochilus*, 33 of *Sylvia communis*, 16 of *Acrocephalus schoenobaenus*, 24 of *Fringilla coelebs*, 7 of *Spinus spinus*, 7 of *Sturnus vulgaris*, 5 of *Parus major*, and 4 of *Parus coeruleus*. Approximately 20,000 red blood cells were screened in each blood film for determining the parasitaemia synchronisation in *Haemoproteus* species-positive blood films, which were processed for microscopic examination using standard protocols [5]. Parasitaemia was considered asynchronous if young gametocytes (size close to the erythrocyte nuclei or less) and mature gametocytes were observed in parallel. An Olympus BX61 light microscope (Olympus, Tokyo, Japan) equipped with an Olympus DP70 digital camera and imaging software AnalySIS FIVE, 2005 Soft Imaging System, Olympus Soft Imaging Solution GmbH, Münster, Germany) was used to examine preparations and prepare illustrations. The statistical analysis was carried out using the ‘Statistica 7’ package. Prevalences of infection were compared by Yates’ corrected Chi-square (χ^2^) test. A *p*-value of ≤0.05 was considered significant.

## 3. Results and Discussion

The following underestimated peculiarities of haemosporidians’ survival in avian hosts were identified during the recent field and experimental observations, which call for deeper and more nuanced research on the parasites’ life cycles and modes of their existence in the wild.

### 3.1. Underestimated Role of Blood Merogony in the Long-Lasting Persistence of Plasmodium Species in Avian Hosts

Recent observations on *Plasmodium* species in experimentally infected birds showed that the long-lasting parasitaemia (from a couple of months to a year and even longer) is not necessarily accompanied with the exo-erythrocytic (or tissue) merogony. In other words, the long-lasting parasitaemia might be supported entirely due to the erythrocytic merogony, without involvement of tissue merogony. Examples are the experimental infections of *P. delichoni* (genetic lineage pCOLL6), *P. homonucleophilum* (pSW2), and *P. relictum* (pSGS1), which were induced in birds by inoculation of infected blood [6,26,27]. The results of these experiments suggest the existence of avian malaria pathogens, which develop like *Plasmodium malariae* in humans [28,29], with long-lasting parasitaemia maintained solely due to erythrocytic merogony. This also implies that the exo-erythrocytic merogony of such avian malaria species might be established mainly by sporozoites, but phanerozoites—a unique tissue stage, which is initiated by erythrocytic merozoites and morphologically look like typical meronts during avian malaria—might not necessarily develop in some parasite species or some avian hosts or during non-natural blood-induced infections. Phanerozoites certainly develop in some blood-induced avian malarial infections [5,30,31], but not in all parasite species [6,26,27]. Experimental observations on natural sporozoite-induced avian malaria infections are needed to finally unravel the mechanisms of formation of phanerozoites, but such experiments remain rare, partly due to unknown vector species of the most-described *Plasmodium* parasites, especially in tropical regions [2]. It also remains unclear if hypnozoites—the sporozoite-induced persisting unicellular stages responsible for relapses—develop during avian malaria [6,32]. There are insufficient experimental data on this issue in all described haemosporidian species. However, unravelling of this question is fundamental for the better understanding of the persistence mechanisms and epidemiology of avian malaria.

### 3.2. Underestimated Role of Gametocytes in the Long-Lasting Survival of Avian Haemoproteus Parasites in Avian Hosts

The role of gametocytes in persistence of haemosporidian infections remains insufficiently understood in avian hosts. Traditionally, it is assumed that gametocytes are short-living life cycle stages, for which lifespan is about a week, so their role in the parasite survival should be secondary in comparison to tissue stages. The latter might persist longer, particularly due to the development of successive generations of tissue meronts [5,31,32,33,34]. However, recent observations suggest that gametocytes of haemoproteids might be an important stage of survival during certain periods of infection due to the following reasons [35].

**Figure 1:**
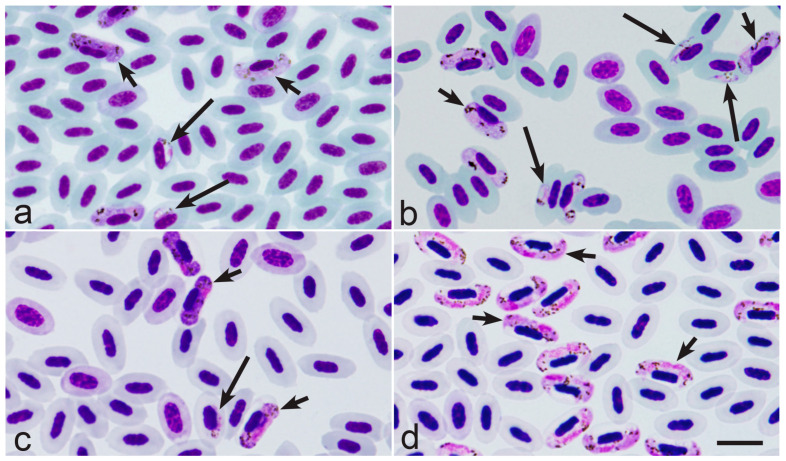
Asynchronous parasitaemia (**a**–**c**) and synchronous parasitaemia (**d**) of *Haemoproteus pastoris* (genetic lineage hLAMPUR1) from the blood of common starling *Sturnus vulgaris* (**a**), *Haemoproteus majoris* (hPARUS1) from the blood of great tit *Parus major* (**b**), *Haemoproteus fringillae* (hCCF3) from the blood of common chaffinch *Fringilla coelebs* (**c**), and *Haemoproteus palloris* (hWW1) from the blood of willow warbler *Phylloscopus trochilus* (**d**). Note that young gametocytes are present together with mature gametocytes during asynchronous parasitaemia (**a**–**c**), but young gametocytes are absent during synchronous parasitaemia, which consists exclusively of mature gametocytes (**d**). Long simple arrows—young gametocytes; short simple arrows—mature gametocytes. Giemsa-stained blood films. Scale-bar: 10 µm.

**Figure 2:**
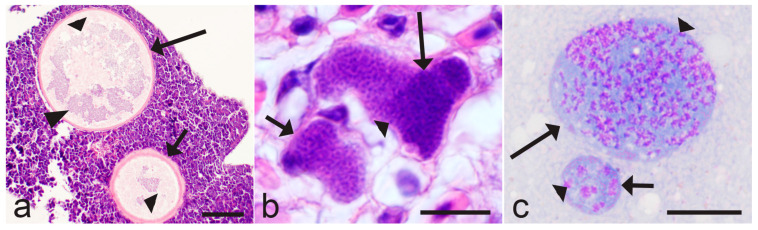
Asynchronous development (**a**–**c**) of tissue stages in avian *Haemoproteus* (**a**,**b**) and *Plasmodium* (**c**) parasites. Uneven maturation of megalomeronts (**a**) and meronts (**b**,**c**) contributes to the asynchronous maturation of merozoites, resulting in development of asynchronous parasitaemia (see Figure 1a–c). Note that young meronts are distinguishable from more advanced meronts due to smaller size (**a**,**c**) and the more prominent cytoplasm and nuclei (**c**). Cytomeres are readily visible in megalomeronts (**a**), but absent in meronts (**b**,**c**). Triangle wide short arrow—young tissues stages; triangle wide long arrow—advanced tissues stages; triangle wide arrowhead—nuclear material; triangle arrowheads—cytomere. Scale bars: (**a**)—100 µm, (**b**,**c**)—10 µm.

In *Haemoproteus* parasites, the merozoites from tissue stages are the only source for the development of gametocytes. The gametocytaemia is usually asynchronous; it consists of young and mature gametocytes occurring in parallel (Figure 1a–c). That is due to the predominantly asynchronous tissue merogony and the asynchronous maturation of merozoites in tissue stages (Figure 2a–c) [5,20,21,34,36,37,38]. The asynchronous tissue merogony can be distinguished in histological preparations due to the different sizes of maturing tissue stages (Figure 2a,c) and the bigger sizes of nuclei and the more prominent cytoplasm in young parasites in comparison to more mature ones (Figure 2c).

Surprisingly, the recent observations show that *Haemoproteus* species parasitaemia in some European far-distance (sub-Saharan) migrating birds is often markedly synchronised in spring after the hosts’ arrival from the African wintering grounds [35]. The synchronised parasitaemia (Figure 1d) consists exclusively of mature gametocytes, with absence of young gametocytes (compare Figure 1a–c with Figure 1d).

A good example of the commonly observed synchronous gametocytaemia is single infections of *Haemoproteus palloris* (Figure 1d) in willow warbles *Phylloscopus trochilus* [35]. Based on the microscopic examination of 38 single infections in this bird species (co-infected samples with other *Haemoproteus* species were excluded), the synchronous parasitaemia was seen in 53% of infected birds. Interestingly, the synchronisation of *Haemoproteus* parasitaemia was observed only during birds’ spring migration and only in some far-distance African migrants as far. For example, during spring migration (May) in Lithuania, the synchronous parasitaemia was common in African far-distance migrants of the Sylviidae (*Sylvia communis*, 52% of synchronous gametocytaemias were seen in the parasite positive individuals, *n* = 33) and Acrocephalidae (*Acrocephalus schoenobaenus*, 56% of synchronous gametocytaemia were seen in the parasite positive individuals, *n* = 16), but was not observed or rare in the most-examined European short-distance migrating birds of the Fringillidae (*Fringilla coelebs*, zero synchronous gametocytaemia was seen in the parasite positive individuals, *n* = 24; *Spinus spinus*, zero synchronous gametocytaemia was seen in the parasite positive individuals, *n* = 7), Sturnidae (*Sturnus vulgaris*, 14% of synchronous gametocytaemia was seen in the parasite positive individuals, *n* = 7), and Paridae (*Parus major* and *Parus coeruleus*, zero synchronous gametocytaemia was seen in the parasite positive individuals in both bird species, *n* = 5 and *n* = 4, respectively). The difference in numbers of observed synchronous parasitaemia in far-distance migrants (87 infected birds examined, among them 46 individuals were with synchronous gametocytaemia) and short-distance migrants (47 infected birds examined, among them only 1 individual with synchronous gametocytaemia) is significant (χ^2^ = 17,73, *p* < 0.0001). These data show that the synchronisation of parasitaemia is more common in some *Haemoproteus* species parasitising far-distance migrants in comparison to short-distance European migrating birds. That was observed in spring when the infected birds arrive to breeding areas after wintering. The following observations can shed light on the explanation of these data.

Surprisingly, the tissue stages were not found in all birds at the stage of synchronous *Haemoproteus* spp. parasitaemia (Figure 1d) during extensive histological observations, which were combined with the genus-specific chromogenic in situ hybridisation testing [35]. Even more, the scar tissues of tissue stages [39] were also not seen during histological examination in the birds with intense synchronous parasitaemia [35]. These data suggest that the exo-erythrocytic merogony obviously terminated in birds some time before their capture and examination in Europe. It is possible that all exo-erythrocytic merogonies completed and merozoites released in the African migrants while they were at wintering grounds before spring migration to Europe. In other words, the synchronised elimination of tissue meronts might occur, resulting in the synchronisation of gametocyte maturation. That is theoretically possible because the maturation of *Haemoproteus* gametocytes occurs within 3–5 days after penetration of merozoites in red blood cells [40]. When the infected birds reach the European breeding sites, all successive tissue merozoites would already reach the stage of mature gametocytes (fully grown ones), resulting in parasitaemia consisting exclusively of mature gametocytes in some bird individuals (Figure 1d). That should contribute to spread of haemoproteosis. This hypothesis coincides with the presence of *Culicoides* vectors at different sites in Europe in spring. Depending on the geographical location, the biting midges are already active between April and the beginning of June in Europe [2,5].

It is essential to point out that the synchronous parasitaemia consisting of the exclusively mature gametocytes was not accompanied with tissue merogony in all tested birds sampled after their arrival from African wintering ground [35]. In other words, the synchronous parasitaemia persisted without tissue merogony. That suggests a relatively long survival of mature gametocytes in the circulation after termination of the tissue merogony. The cease of exo-erythrocytic development and the long-lasting survival of gametocytes in infected red blood cells of avian hosts should be beneficial both to parasites and birds, so might be supported by natural selection due to the following reasons.

First, the far-distance migrating birds cross prominent ecological barriers during migration (Sahara Desert, Mediterranean Sea, mountains, etc.), and such flight is energy-consuming [41,42,43,44]. The clearance of active exo-erythrocytic merogony before migration would minimise damage of bird internal organs by tissue stages; this might be advantageous both for the avian health and the parasites’ survival. The damage caused by tissue stages of haemoproteids can be significant [18,21,34,36,37,38,45,46]. If infected birds survive, the parasites survive in the host as well.

Second, the long-term presence of mature gametocytes in the circulation is functional for parasite survival because it provides opportunities for the infection of vectors and the transmission on stopover sites just after arrival to breeding grounds, implying a ‘clever’ strategy of pathogen existence. In particular, the observed fully grown gametocytes were viable at the stage of the parasitaemia synchronisation because they readily exflagellated and produced ookinetes in vitro [35].

Given the average lifespan of uninfected avian erythrocytes of about a month [47], it is possible that the longtime surviving gametocytes might be the main active parasite stage in some far-distance migrating birds when the exo-erythrocytic merogony is markedly reduced or eliminated. Even more, it is also possible that gametocytes might increase the lifespan of infected erythrocytes because that would be beneficial for transmission. The latter issue remains non-investigated in wildlife haemosporidian parasites but would be not surprising because the experimental observations showed that malaria parasites were able to adjust the sex ratio of gametocytes in infected animals depending on needs of transmission [48,49].

It is possible that tiny *Haemoproteus* parasites ‘predict’ migratory strategy of avian hosts and adjust their development in relation to the host’s life. Experimental observations are needed to unravel the role of gametocytes in long-term survival of some *Haemoproteus* species during energy-consuming periods of host life. To evaluate this issue, the following two groups of haemoproteids can be selected for experimental observations and comparison during spring migration in Europe: first, the single infections of specific parasite species infecting far-distance migrating birds, which cross big ecological barriers (deserts, water bodies, mountains, etc.) during migratory flight; second, the single infections of specific parasite species infecting short-distance migrants, who do not cross big ecological barriers, so theoretically could tolerate infections better due to the permanent availability of stopover sites where they can feed and rest during migration [43]. Based on the available information, it is predictable that the synchronous cease of tissue merogony resulting in the synchronous parasitaemia of exclusively mature gametocytes might be biological features of some haemoproteid species infecting the first group of migrating birds during certain periods of the life cycle. Based on the available data [35], *H. palloris* in willow warbler and species of *Haemoproteus* infecting short-distance migrants (Figure 1a–c) can be recommended for such research in Europe. It is worth mentioning that some effort might be needed to recognise the synchronised parasitaemia in naturally infected birds due to possible co-infections of several *Haemoproteus* species, which might be at different stages of gametocyte development in the same host individuals. This possibility should be considered during microscopic examination. Theoretically, multiple infections of the same parasite species might also contribute to the observed parasitaemia asynchrony. However, it remains unclear how often such infections occur in wildlife due to the difficulties in distinguishing multiple acquired infections of the same parasite lineage both by microscopic examination and traditional PCR-based testing [2].

### 3.3. Underestimated Haemoproteus Infections Due to Slow Maturation of Megalomeronts

**Figure 3:**
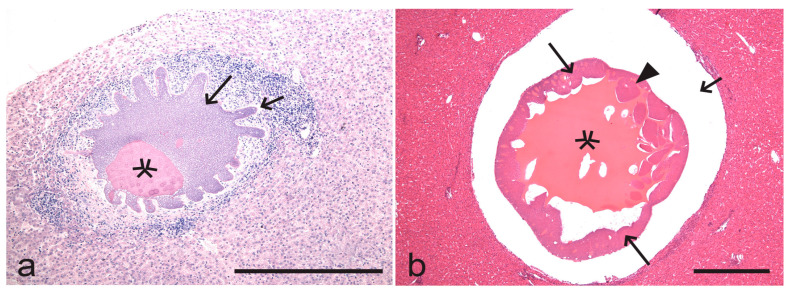
Mature megalomeronts (merocysts) of *Hepatocystis semnopitheci* (unknown genetic lineage) in the liver of southern pig-tailed macaque *Macaca nemestrina* (**a**) and *Hepatocystis taiwanensis* (unknown genetic lineage) in the liver of Formosan rock macaque *Macaca cyclopis* (**b**). Note the big size of the merocysts, which possess large vacuoles containing eosinophilic fluid, the cytomeres, and the numerous developing merozoites. Merocysts are covered with undifferentiated thick envelopes. The outline of the *H. semnopitheci* merocyst (**a**) is extraordinary lobulated. Simple wide short arrows—envelope; stars—vacuoles; triangle arrowheads—cytomere; simple wide long arrows—merozoites. Formalin-fixed haematoxylin-eosin (H&E)-stained (**a**) and Carnoy-fixed H&E-stained (**b**) histological sections. Scale bars: 500 µm.

Megalomeronts (Figure 2a) are the predominate tissue stages in some *Haemoproteus* parasites [5,18,39,50]. Recent observations recognised *Haemoproteus* lineages that develop only megalomeronts during the exo-erythrocytic merogony [36,37,46,51,52,53]. Due to the often large size (up to 500 µm and even bigger in diameter) and the complicated cytomere structure, the growth and maturation of the megalomeronts might take a long time, which remains non-estimated in all *Haemoproteus* species. Interestingly, the huge size megalomeronts (merocysts), which have the complex structure and might be about 1 mm in diameter, develop in *Hepatocystis* species (Figure 3a,b). The tissue stages of these parasites develop and mature slowly. For example, the estimated maturation time of *Hepatocystis kochi* is within 2 months [10,54]. This long-term maturation is expected in megalomeronts of avian *Haemoproteus* species as well because their diameter and complex structure (Figure 2a) are comparable with merocysts (Figure 3a,b) [10,36,37,38,46,51]. The biggest *Haemoproteus* megalomeronts are at least 10 times the diameter of tissue meronts in avian *Plasmodium* parasites; the latter do not produce cytomeres (Figure 2b,c) and mature usually within 1–2 weeks or even faster [5,10].

It is difficult to rule out that the long maturation time of megalomeronts and resulting slow development of infective merozoites might occur in *Haemoproteus* species as is the case in *Hepatocystis* parasites. In other words, gametocytes might be absent in *Haemoproteus* species-infected birds containing the parasites at a stage of immature megalomeronts, as has been reported in *Hepatocystis* species [10]. If parasitaemia is absent, the microscopic examination and PCR-based testing would be negative as well because both diagnostic tests rely mainly on the presence of gametocytes in the circulation [2,17,18,19]. That might result in biased conclusions about absence of infections in the infected birds at a study site.

For example, *Haemoproteus pastoris*, *H. payevskyi*, *H. nucleocondensus*, and *H. hirundinis* are prevalent blood parasites of common starlings *Sturnus vulgaris*, common reed warbler *Acrocephalus scirpaceus*, great reed warblers *Acrocephalus arundinaceus*, and common house martin *Delichon urbicum*, respectively, in Europe. Interestingly, these pathogens are prevalent in adult birds after arrival from wintering grounds in spring but are not found in juveniles of the same species at bird breeding grounds before autumnal migration [5,55,56,57]. It was speculated that transmission of all these *Haemoproteus* infections occurs only outside bird breeding areas (at wintering grounds). However, this epidemiological conclusion contradicts the experimental observations, which showed that the susceptible vectors (*Culicoides* biting midges) of *H. pastoris*, *H. nucleocondensus*, and *H. hirundinis* are present in northern Europe, and the transmission theoretically is possible [58,59]. It is probable that these parasites are transmitted in Europe, and they initiate the development in juveniles of the corresponding bird species. However, these infections maintain at stage of developing immature megalomeronts, for which maturation completes when the birds migrate from the breeding grounds (at the end of European summer) and reach wintering areas where the parasitaemia appears. This is theoretically possible because megalomeronts were found in adults of common starlings and common house martins [46,53].

To make sure that juveniles of the common starlings, common reed warblers, great reed warblers, common house martins, and some other common birds are truly free of haemoproteids at European breeding grounds, the extensive examination of organs is needed in juveniles of the corresponding species, aiming the search for growing (immature) megalomeronts. Naturally dead wild bird individuals might be used for such research because the birds sometimes die when colliding with buildings, lighthouses, windmill turbines, and other constructions. Citizen science-based surveys can be helpful [52]. The dead birds can be collected and stored at –20 °C for later histological and parasitological analysis. Due to restrictions on experimental observations with wild animals, that seems to be the easiest way to address the long-existing puzzle about the pattens of transmission of *H. pastoris*, *H. payevskyi*, *H. nucleocondensus*, *H. hirundinis,* and other haemoproteids in Europe. This knowledge is important for a better understanding of the haemoproteosis epidemiology. Recent discoveries of *Haemoproteus* megalomeronts in European passerine birds [36,37,38,46,51,52,53] provided a background to call for the testing of this hypothesis.

### 3.4. What Factors Drive the Narrow Specificity of Haemoproteus Parasites in Birds?

Molecular genetic studies showed that the majority of *Haemoproteus* cytb lineages are vertebrate-host-specific; they predominantly occur in certain bird species or a few closely related species [2]. Strangely, nearly each bird species often is infected with a different *Haemoproteus* genetic lineage, and the number of the lineages might be bigger than number of their avian host species [2,60,61]. Some *Haemoproteus* lineages were reported in birds of different families or even orders, but such genetic reports usually were not supported with observation of blood stages (gametocytes) of the corresponding parasites [19,62]. In other words, these likely are abortive (on sporozoite or tissue stages) haemosporidian infections, which do not complete development and are the dead ends of transmission in the PCR-positive avian hosts [20].

The mechanisms of the narrow specificity of *Haemoproteus* parasite lineages in birds remain unclear, and there is no convincing explanation of this phenomenon. A vector factor can hardly explain restriction of certain parasite lineages to a narrow group of avian hosts. Ornithophilic *Culicoides* species—the vectors of most *Haemoproteus* parasites—often bite a broad range of bird species and the same species of biting midges are susceptible to many *Haemoproteus* species [5,50,63,64,65,66,67]. That provides opportunities for the broad dissemination of infective stages (sporozoites) among different bird species living in the same environment. The internal factors of the vertebrate host–parasite interactions should have a priority in development of the narrow specificity of *Haemoproteus* parasites in avian hosts. How to explain the restriction of certain parasite species to a narrow group of avian hosts from this point of view? The following data are worth considering.

**Figure 4:**
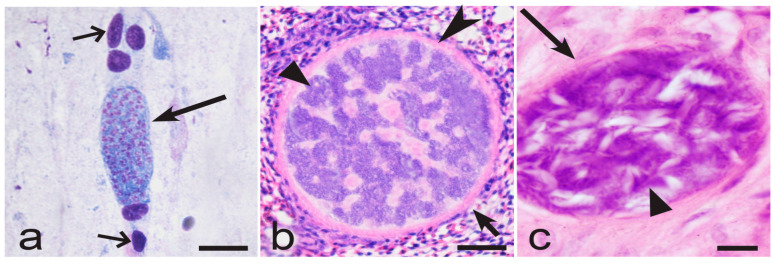
Maturing tissue stages of *Plasmodium gallinaceum* (genetic lineage pGALLUS01) in brain of domestic chicken *Gallus gallus domesticus* (**a**), *Haemoproteus majoris* (hPARUS1) in lungs of great tit *Parus major* (**b**), and *Haemoproteus columbae* (hCOLIV01) in lungs of rock dove *Columba livia* (**c**). Note that the tissue meront of *Plasmodium* parasite does not contains cytomeres; the parasite nuclei are scattered homogenously in the cytoplasm (**a**). On the opposite, both megalomeront (**b**) and meront (**c**) of *Haemoproteus* parasites contain numerous variously shaped cytomeres, which overfilled with nuclear material. Long simple arrows—meronts; short simple arrow—megalomeront; triangle arrowheads—cytomeres; simple arrowheads—capsular-like wall of megalomeront; simple wide short arrows—nuclei of erythrocytes in brain capillary. Formalin-fixed haematoxylin-eosin (H&E)-stained histological sections. Scale bars: (**a**,**c**)—10 µm; (**b**)—50 µm.

Interestingly, the narrow specificity of *Haemoproteus* lineages in birds is opposite to that of the closely related *Plasmodium* parasites, which have a broader range of competent avian hosts. The same malaria parasite lineages often complete life cycles and produce vector-invasive gametocytes in birds belonging to different genera, families, and even orders [5,30,31,61,68,69,70]. The differences in host specificity of these two groups of closely related haemosporidians might be due to the differences in development of their tissue stages. The latter occur obligatory both during avian malaria and haemoproteosis, but the maturation process is distinct in these parasites. The host cells of tissue stages remain insufficiently investigated in bird *Haemoproteus* and *Plasmodium* parasites on species levels, and they probably are mostly restricted to non-specialised reticular and endothelial cells [5,10,19,30,32,50]. However, the major structure of developing tissue stages in these two groups of avian haemosporidians is strikingly different. In avian *Plasmodium* species, only meronts (never megalomeronts) develop, and the formation of merozoites usually occurs without development of cytomeres (Figure 2c and Figure 4a). In other words, the tissue merogony in avian *Plasmodium* parasites is a relatively straightforward process, which is comparable with erythrocytic merogony in this regard. In contrast, the maturation of tissue stages in *Haemoproteus* species occurs via the obligatory stage of cytomeres’ formation both in megalomeronts (Figure 2a and Figure 4b) and meronts (Figure 4c) [5,20,34,36,37,38,45,46,50,52,53]. Mainly, each developing tissue stage splits into a multitude of multinuclear cytomeres, which either remain interconnected or separated from each other by the plasmalemma and the content of the parasitophorous vacuole. Merogony within the cytomeres leads to development of merozoites [5]. Thus, the more complicated nuclear division process and the transformation of content of tissue stages occur during the exo-erythrocytic merogony of *Haemoproteus* species in comparison to *Plasmodium* spp. Tissue stages of avian *Haemoproteus* and *Plasmodium* parasites are distinguishable based on the presence of cytomeres (compare Figure 4a with Figure 4b,c), which is a helpful diagnostic characteristic for identification of these genera in histological section during co-infections.

**Figure 5:**
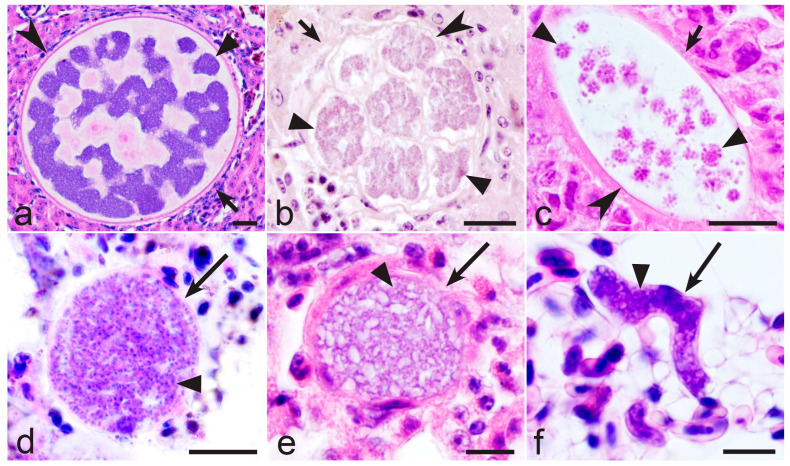
Developing tissue stages of *Haemoproteus majoris* (genetic lineage hPARUS1) in kidneys of great tit *Parus major* (**a**), *Haemoproteus passeris* (unknown lineage) in liver of house sparrow *Passer domesticus* (**b**), *Haemoproteus hirundinis* (hDELURB2) in pectoral muscles of common house martin *Delichon urbicum* (**c**), and different maturation stages of *Haemoproteus attenuatus* (lineage hROBIN1) in lungs (**d**,**f**) and kidneys (**e**) of European robin *Erithacus rubecula* (**d**) and thrush nightingale *Luscinia luscinia* (**e**,**f**). Note that maturing megalomeronts (**a**–**c**) and meronts (**d**–**f**) contain numerous variously shaped cytomeres, for which morphology is species-specific and distinct in different parasite species. Short simple arrows—megalomeronts; long simple arrows—meronts; triangle arrowheads—cytomeres; simple arrowheads—capsular-like walls of megalomeronts. Formalin-fixed haematoxylin-eosin (H&E)-stained histological sections. Scale bars: (**a**)—20 µm; (**b**,**c**)—50 µm; (**d**–**f**)—10 µm.

The complicated mode of maturation of tissue stages might be a driver of vertebrate host specificity of *Haemoproteus* parasites due to the remarkably different process of cytomere formation and the markedly different morphology of the cytomeres in different *Haemoproteus* species (Figure 5a–f). These differences are parasite species-specific [20,34,36,37,38,46,53], so should be determined genetically, but the latter knowledge is absent with regard to tissue stages of haemoproteids on species levels. It is possible that formation of cytomeres in certain parasite lineages require specific host physiological requirements, for which a lack of might limit the opportunities for the maturation of tissue stages in incidental (‘wrong’) avian hosts. In other words, if vectors (*Culicoides* biting midges or Hippoboscidae louse flies) inoculate sporozoites of *Haemoproteus* parasites in the ‘wrong’ avian host, the exo-erythrocytic development might be either eliminated initially or initiated, but then arrested, resulting in abortion on tissue stages, as recently was documented using chromogenic in situ hybridisation methods [20]. As a result, only certain parasite lineages could complete development and produce gametocytes in certain bird species, resulting in restriction of parasite lineages to the closely related host species and the narrow vertebrate host specificity.

The morphological diversity of tissue stages and cytomeres in *Haemoproteus* parasites is incredible (Figure 2a, Figure 4b,c and Figure 5a–f); its complexity is comparable with *Hepatocystis* pathogens (Figure 3a,b) and the related *Culicoides*-transmitted haemosporidians in mammals [10,54,71]. In other words, the phenotypic characters of tissue stages of haemosporidian parasites are not only of taxonomical importance [20,36,37,53,72] but also might be drivers of host specificity, calling for targeting delicate research aiming better understanding mechanisms of host–parasite relationships during the exo-erythrocytic development. Such knowledge is absent in avian haemosporidian parasites.

It is worth noting that the abortive *Haemoproteus* infections are dead ends regarding transmission due to inability to form gametocytes, but such infections might be virulent and even lethal in birds [21,45,73,74]. Abortive haemosporidian infections are common in wildlife [18,19,20,21,22,23,24,62]. The identification of molecular mechanisms that are responsible for the abortion of development during tissue stages might indicate new methods for control of haemosporidioses. The diverse and widespread avian *Haemoproteus* species can be used as model organisms for search of molecular markers, which are responsible for blockage of exo-erythrocytic development in haemosporidian parasites.

## 4. Conclusions

The general lines of life cycles are well defined in all families of haemosporidian parasites; however, the details of development and survival remain insufficiently understood in wild vertebrates. The recent findings suggest that the lives of haemosporidian pathogens are more complex than assumed currently. Opposite to the orthodox opinion, the new discoveries suggest a more significant role of blood stages in the life of avian haemosporidians. Particularly, both the role of erythrocytic merogony in long-term survival of avian *Plasmodium* species and the role of sexual stages (gametocytes) in survival of *Haemoproteus* parasites in vertebrates seem underestimated. In other words, the blood stages not only perform the functions of multiplication (erythrocytic meronts) and sexual potency (gametocytes), as usually considered, but also contribute to prolonged survival (persistence) in avian hosts during certain stages of infection. Additionally, the tissue merogony plays not only the function of asexual multiplication in *Haemoproteus* parasites but also might be a driver of narrow vertebrate host specificity due to the complicated maturation of tissue stages, which involves the obligatory complicated process of cytomere formation. The structure of developing cytomeres is species-specific and phenotypically distinct in different *Haemoproteus* parasites, so should be genetically determined. Haemosporidian parasites closely adjust their development to the life of avian hosts; however, the delicate mechanisms responsible for the host–parasite interactions remain unclear, calling for targeting experimental research, which remains at the infancy stage in these remarkably diverse wildlife pathogens.

## Data Availability

The original contributions presented in the study are included in the article. Further inquiries can be directed to the first author.
